# Development of Smartphone Applications for Nutrition and Physical Activity Behavior Change

**DOI:** 10.2196/resprot.2205

**Published:** 2012-08-22

**Authors:** Lana Hebden, Amelia Cook, Hidde P van der Ploeg, Margaret Allman-Farinelli

**Affiliations:** 1 School of Molecular BioscienceThe University of SydneySydneyAustralia; 2 Sydney School of Public HealthThe University of SydneySydneyAustralia

**Keywords:** cellular phone, young adult, primary prevention, lifestyle, health behavior

## Abstract

**Background:**

Young adults (aged 18 to 35) are a population group at high risk for weight gain, yet we know little about how to intervene in this group. Easy access to treatment and support with self-monitoring of their behaviors may be important. Smartphones are gaining in popularity with this population group and software applications (“apps”) used on these mobile devices are a novel technology that can be used to deliver brief health behavior change interventions directly to individuals en masse, with potentially favorable cost-utility. However, existing apps for modifying nutrition or physical activity behaviors may not always reflect best practice guidelines for weight management.

**Objective:**

This paper describes the process of developing four apps aimed at modifying key lifestyle behaviors associated with weight gain during young adulthood, including physical activity, and consumption of take-out foods (fast food), fruit and vegetables, and sugar-sweetened drinks.

**Methods:**

The development process involved: (1) deciding on the behavior change strategies, relevant guidelines, graphic design, and potential data collection; (2) selecting the platform (Web-based versus native); (3) creating the design, which required decisions about the user interface, architecture of the relational database, and programming code; and (4) testing the prototype versions with the target audience (young adults aged 18 to 35).

**Results:**

The four apps took 18 months to develop, involving the fields of marketing, nutrition and dietetics, physical activity, and information technology. Ten subjects provided qualitative feedback about using the apps. The slow running speed of the apps (due to a reliance on an active Internet connection) was the primary issue identified by this group, as well as the requirement to log in to the apps.

**Conclusions:**

Smartphone apps may be an innovative medium for delivering individual health behavior change intervention en masse, but researchers must give consideration to the target population, available technologies, existing commercial apps, and the possibility that their use will be irregular and short-lived.

## Introduction

Across developed countries, the average person owns 1.18 mobile phones with this number continuing to rise [1]. Much of this growth has been in smartphone ownership (mobile phones with computer and Internet capabilities); there were more than 490 million shipments of smartphones globally in 2011 compared to approximately 300 million in 2010 [2]. The growth in the smartphone market has been concentrated in young adults, especially in the United States, with 62% of mobile phone users aged 25-34 owning a smartphone in 2011, an increase from 41% in 2010 [3].

The development of smartphones has led to a proliferation of smartphone software applications (“apps”), which are programs able to run on these mobile devices. From a public health perspective, smartphone apps can potentially enhance the delivery of health behavior change interventions to individuals en masse and result in favorable cost-utility. Despite this, researchers to date have largely developed apps to intervene in the clinical care setting for patient self-management, whereby a patient monitors themselves and receives therapeutic feedback [4,5], or for real-time therapy where no self-reported data is required from the patient [6,7]. The commercial sector has developed numerous apps for weight loss that include information on nutrition and physical activity, although the majority are based on calorie counting approaches and may not always reflect best practice guidelines for weight management [8].

In most Western countries, young adults (ages 18 to 35) are a population group at high risk for becoming overweight or obese [9,10]. For example, in the US Coronary Artery Risk Development in Young Adults (CARDIA) cohort, it was reported that females gained an average 0.7 kg and males gained an average 0.8 kg, each year [11]. There are four key lifestyle behaviors that appear to play an important role in the etiology of weight gain in this population group. These behaviors include a decline in physical activity [12,13], excessive intake of high-fat take-out (fast food) meals [14], over-consumption of sugar-sweetened drinks [15,16], and an inadequate consumption of fruit and vegetables [17]. However, there is limited evidence to inform what method of intervention might be effective for preventing weight gain in this group [18], although easy access to treatment and providing support for planning and self-monitoring behavior may be important [19,20]. Hence, we embarked on building a series of smartphone apps to assist young adults in forming healthier lifestyle habits. This paper describes the process of developing four separate smartphone apps and discusses our insights from this process.

## Methods

The development process consisted of four stages: (1) deciding on the specifications, (2) selecting the platform, (3) creating the design, and (4) testing the prototypes.

### Stage 1: Deciding on the Specifications

The first stage of this process involved defining the purpose of each app. This required specifying the relevant public health guidelines to inform the goals for behavior change, the specific strategies for behavior change, the visuals or graphic design, and the potential data to be collected.

**Figure 1:**
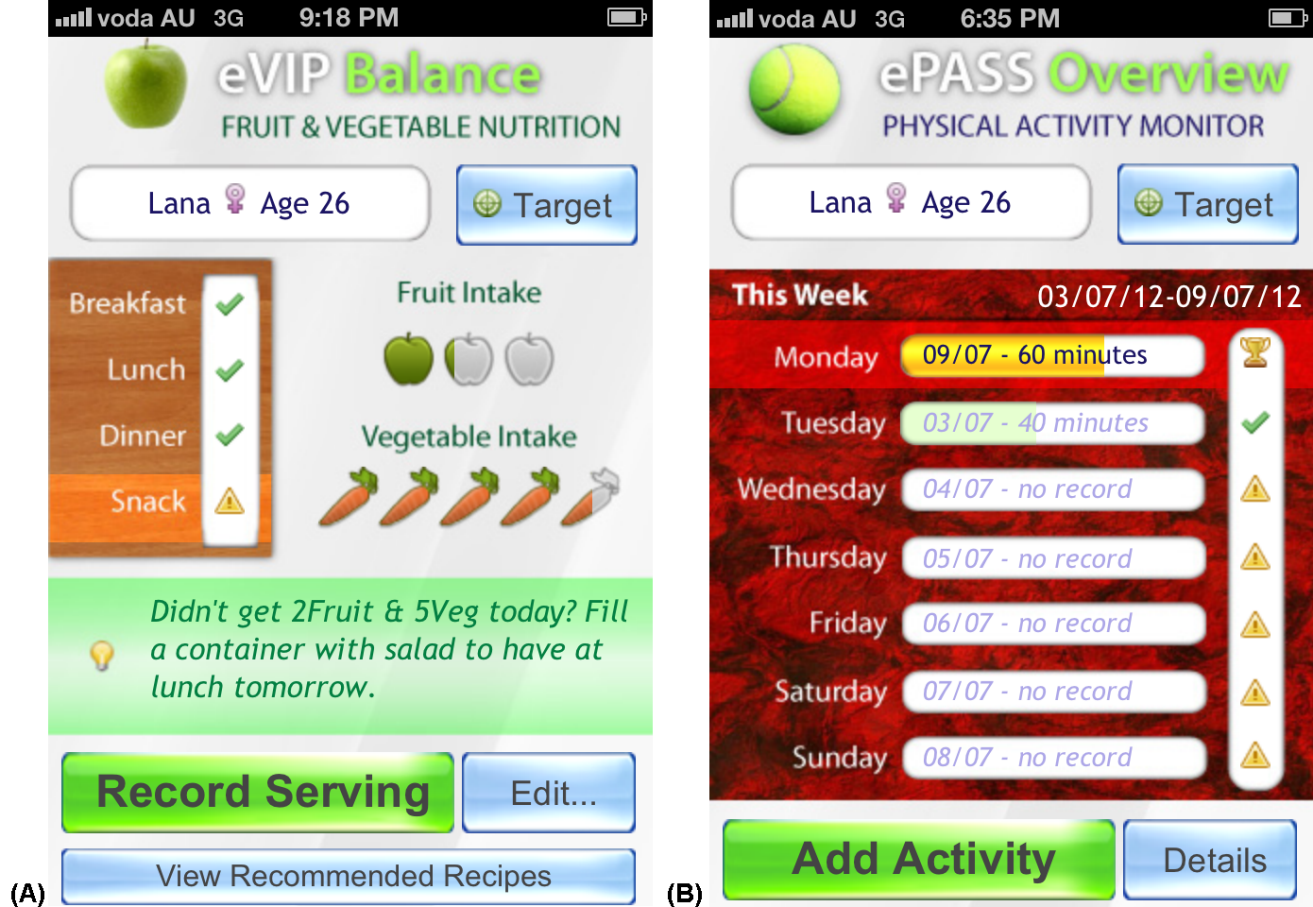
Screenshots taken on an iPhone device illustrating the user interface: home screen provides an overview of fruit and vegetables recorded in eVIP (A) or physical activity recorded in ePASS (B), in light of reference population health guidelines.

The fundamental purpose of the apps was to support change in the lifestyle behaviors identified. To assist young adults with improving their dietary habits, goals for these behaviors had to be defined to create rules about what is adequate [21,22]. Relevant public health guidelines were consulted for physical activity levels [23,24], intake of fruit and vegetables [25], and recommended limits for take-out meals and sugar-sweetened drinks [26,27]. For example, the physical activity app (ePASS) included a “good health” target of 30 minutes of moderate level physical activity daily based on the World Health Organization’s physical activity guidelines of at least 150 minutes of aerobic activity per week for adults [24], and a “healthy weight” target of 60 minutes of moderate level physical activity daily based on expert consensus that up to 60 minutes of moderate activity daily is required to prevent unhealthy weight gain [23] (see Figure 1B). The fruit and vegetable app (eVIP) provided users with a graphical display of the number of fruit and vegetable servings they recorded out of the two servings of fruit and five servings of vegetables recommended by the Australian Government Department of Health and Aging (see Figure 1A) [25]. The sugar-sweetened drinks app (eSIYP) presented users with a color display of their total energy, sugar, and alcohol intake from all drinks consumed, in which green, orange, and red indicated the “ideal,” “acceptable,” and “too much” threshold levels of intake, respectively. These threshold levels were based on nutritional expert opinion of the recommended limits on consumption of added sugars, as per the Australian Dietary Guidelines [26]. The take-out food app (eTIYP) also presented users with a color display of the average energy and fat content of take-out meals, in which green indicated acceptable intake and red indicated excessive intake, equating to ≤30% and >30% of the dietary intake recommended by the Australian National Health and Medical Research Council and the New Zealand Ministry of Health, respectively, according to age and gender [27].

In terms of behavior change strategies, young adults often lack the self-regulatory skills, such as self-monitoring and planning, required to adopt and maintain healthy behaviors [20]. Self-regulation was fostered in each app by providing users with a platform to create daily entries of their behavior (eg, physical activities performed or vegetables consumed) from which they were provided daily or weekly summaries of their reported behavior, in reference to public health guidelines, to enable their monitoring of and planning around these behaviors. Providing feedback that is personally relevant may also be an important strategy for changing young adults’ behaviors [18]. Encouragement from success of attaining a goal and social persuasion can enhance self-efficacy, and has been identified as important in achieving behavior change, particularly during busy or stressful situations [28,29]. All apps provided motivational tips as a source of positive encouragement that would assist the young adults in creating more positive beliefs around their ability to change their behavior (eg, “You can split up your exercise target into as little as 15-minute bursts”). These tips were also tailored to users’ self-reported behavior. For example, if a user’s reported physical activity did not meet recommended guidelines, they were shown a relevant motivational tip (ie, “Plan exercise in advance and write it down if you can—try phone reminders”).

Consideration was also given to the graphic design and how this might influence behavior. The behavior-image model suggests that through processes of social- and self-comparison, individuals will compare themselves to similar human images and create projections of themselves possessing the desired characteristics of the humans in those images [30]. This process is referred to as “self-reevaluation” in the Transtheoretical Model, which uses healthy role models and imagery to assist ones progression from contemplating behavior change to preparing for changing behavior [31]. Hence, in the apps we used images of young adults who were performing the target behaviors (eg, riding a bike or drinking water) and possessing desirable characteristics of a normal healthy appearance and lifestyle, to motivate users toward changing the target behaviors. Similarly, we displayed healthier foods and drinks, rather than “junk” foods, to model these foods as ideal for consumption. All images were purchased from a commercial graphics company to avoid potential breaches in copyright.

To facilitate future research, we enabled the following data items to be exported: user identification (ID), log-in ID, sex, age, and the date and time of log-ins into each app. Additional information that was able to be exported from each app included: physical activities performed and their duration; drinks consumed and their volume, total sugar, alcohol, and energy content; energy and fat content of take-out meals, the restaurant where the meal was consumed, and the contents of the meal; and the number of servings and types of fruit and vegetables consumed. This data could be exported into comma-separated value files from our relational database (described later), which could then be exported into statistical software for further analysis.

### Stage 2: Selecting the Platform

Traditionally, apps are developed for one specific operating system (*native *app), such as iOS, Android, Windows, Symbian, and BlackBerry, or developed as *Web-based *apps. Native apps run locally on a smartphone’s operating system in a way that is analogous to programs running on a desktop computer. Web-based apps run like a Web page, whereby the app operates on an external server and the user accesses the app through the Web browser on their mobile device. Due to these functional differences, Web-based apps may be used on all smartphones regardless of the operating system as long as the user has Internet access; however, there is less opportunity to utilize the existing hardware built into the phone (eg, the camera, geopositioning, or calendar). In our case, we developed Web-based apps because we did not require the use of existing hardware on the phone and we wanted to enable downloading of data recorded by users and allow the apps to be used on multiple operating systems and through the Internet for those who did not own a smartphone. However, Web-based apps are increasingly becoming able to perform like native apps with an offline mode that can be accessed without Internet connectivity. Similarly, there is increasing potential for native apps to possess Internet connectivity, enabling the user to download updates and data from the user to be uploaded to a server. This emphasizes the dynamic nature of mobile technologies and why the type of platform selected should be discussed with an information technology specialist.

### Stage 3: Creating the Design

To enable users to record their behavior, each app had to be linked with the relevant data for that behavior. The following data items were included for each app: 

1. ePASS: type of activity (ie, gym, sports, recreational, or housework) and the intensity (ie, moderate vs vigorous) of 91 unique activities, where “moderate” was defined as a metabolic equivalent of task (MET) value of 3-6 and “vigorous” was defined as > 6, derived from the compendium of physical activities [32].

2. eVIP: serving size equivalents for 48 types of fruit and 61 vegetables, where 1 serving was equivalent to 150 g of fruit or 75 g of vegetables [26] (eg, 0.5 cups chopped or 4 spears of cooked asparagus are both equivalent to 1 serving of vegetables).

3. eTIYP: total energy and fat content of 504 take-out food and drink menu items.

4. eSIYP: drink category (eg, waters, vitamin waters, hot chocolate, tea/coffee, alcohol, soft drinks, sports drinks, cordials, fruit juices, fruit drinks, flavored milks, and milkshakes) and the total energy, sugar, and alcohol content of 114 unique drinks.

Nutrient composition data for eTIYP and eSIYP were sourced from the Australian government food and nutrient database, NUTTAB [33]. The foods listed in this database were chemically analyzed or, when unavailable, sourced from food manufacturer nutrient label data which may or may not be based on chemical analysis.

These data items were all contained within one relational database, which is essentially a database where the data items (or variables) are arranged into a series of tables with each table representing a different aspect or “relation” in the data. For each app, there were 1-2 tables containing the behavioral data. For example, the eVIP app required one table for the types of fruit and vegetables, such as “eggplant/aubergine (cooked),” and a second table listing the portion sizes, such as “0.5 cups diced,” “3 thin slices,” or “1 thick slice,” with their respective serving equivalents of 1 serving, 1 serving and 0.5 servings. An additional table was included for each app containing the motivational tips and another table contained user details (ie, name, age, gender, log-in ID, and user ID). The relational database management system software, MySQL (Oracle Corporation, Redwood Shores, California, United States), was used to access and query data items contained within the relational database using structured query language (SQL). For example, if a user logged into the eVIP app at lunchtime, SQL was used to identify a motivational tip about including fruit or vegetables at the lunch meal to present to the user from the motivational tips table in the relational database for the eVIP app.

**Figure 2:**
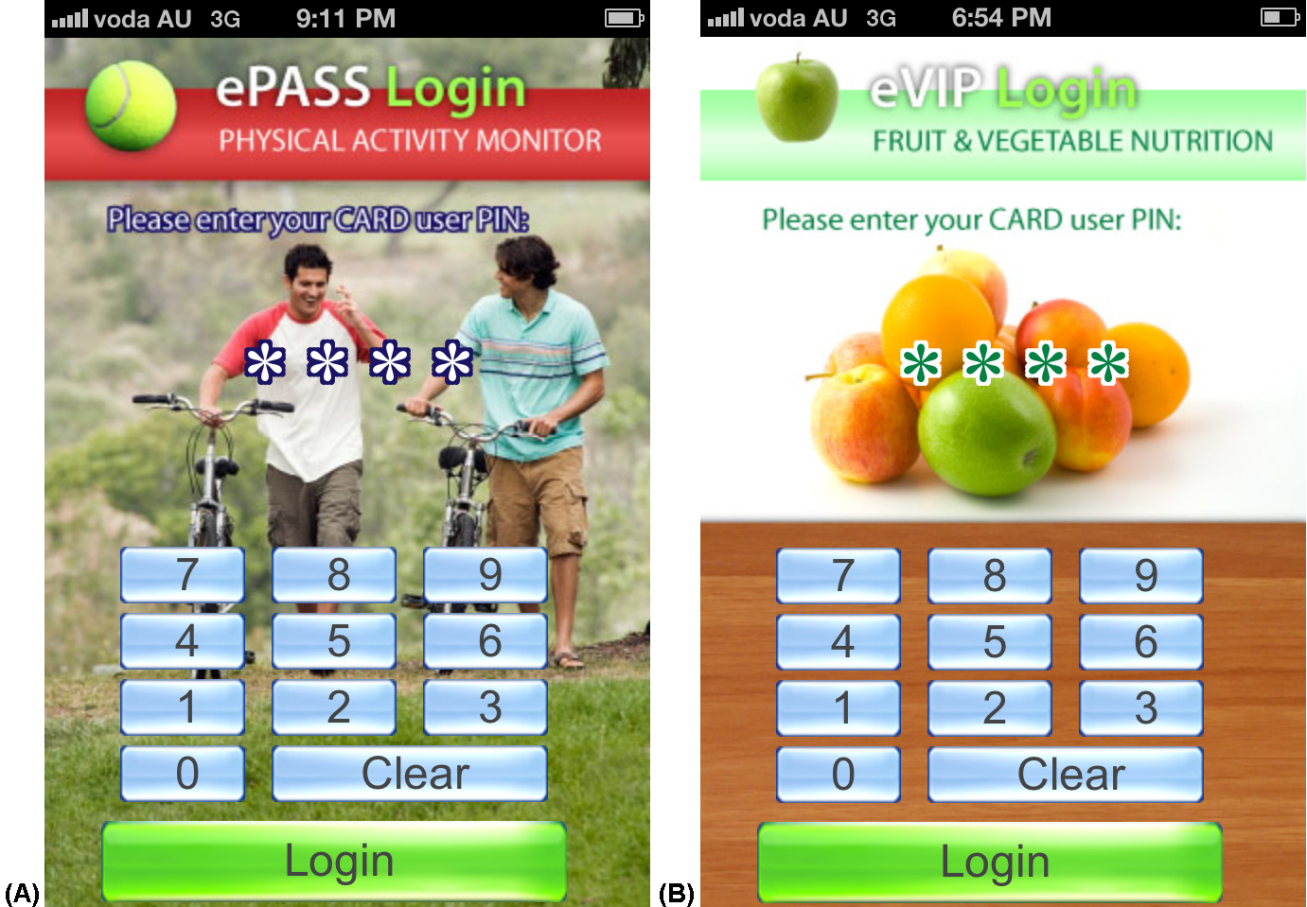
Screenshots taken on an iPhone device illustrating the user interface: log-in screens where users enter their unique ID for user privacy and protection of intellectual property.

**Figure 3:**
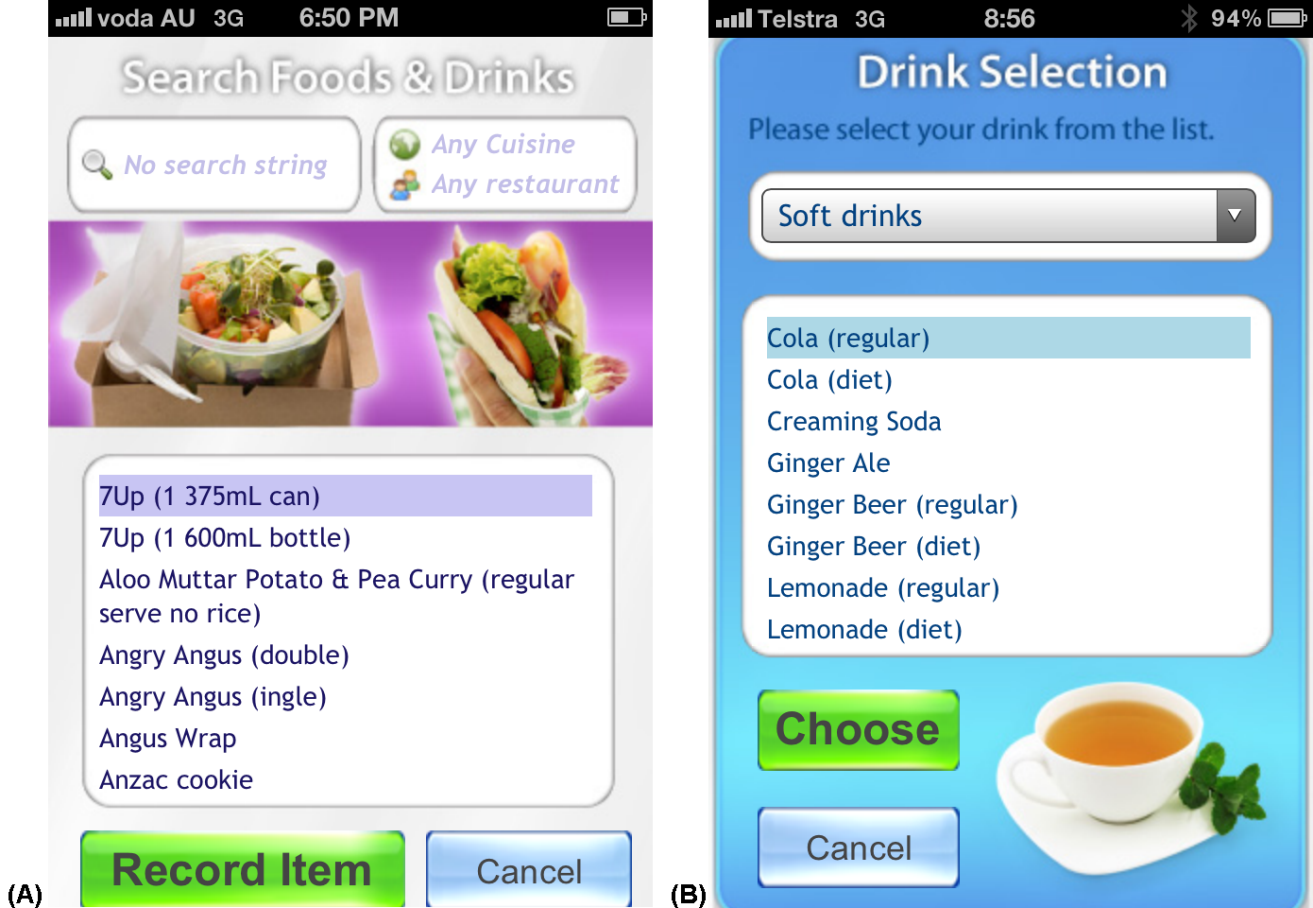
Screenshots taken on an iPhone device illustrating the user interface: users may record their take-out meals in eTIYP (A) or their drinks in eSIYP (B).

**Figure 4:**
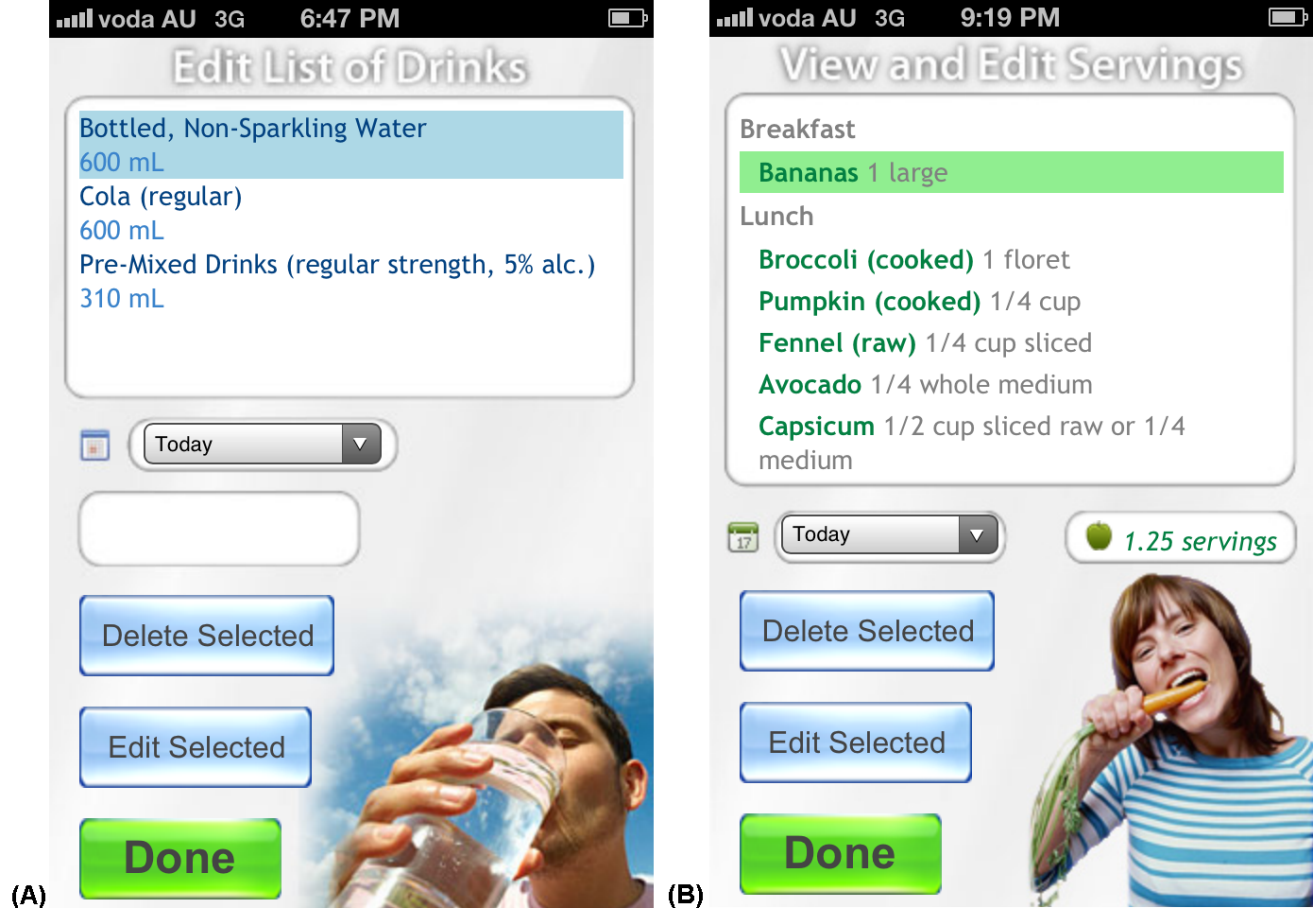
Screenshots taken on an iPhone device illustrating the user interface: users may review or edit their drinks in eSIYP (A) or their fruit and vegetable intake in eVIP (B).

**Figure 5:**
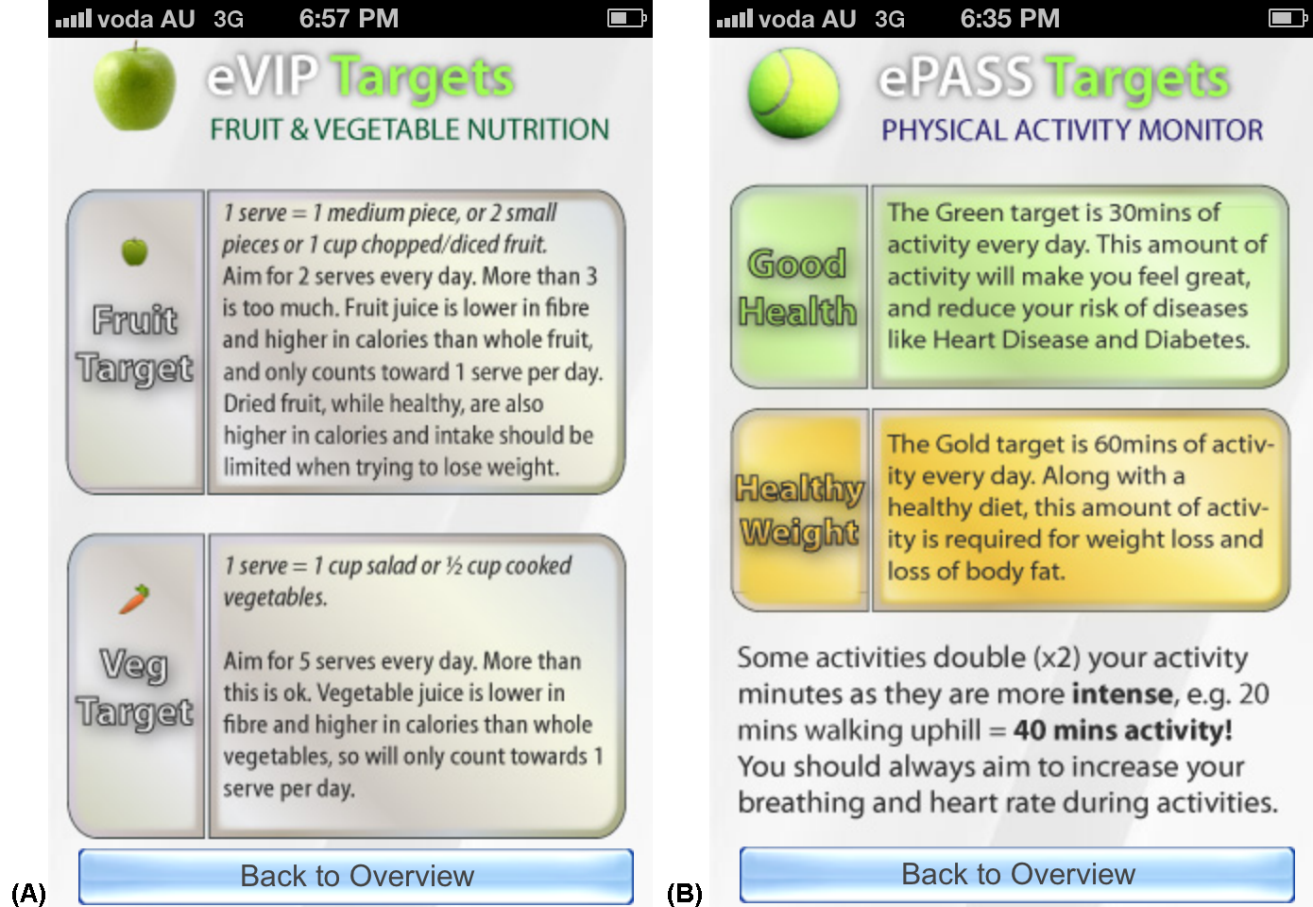
Screenshots taken on an iPhone device illustrating the user interface: targets screen provides the user details about the reference guidelines.

Programming for the apps was written with Python programming language software (Python Software Foundation, Wolfeboro Falls, New Hampshire, United States) to communicate with the relational database and generate the user interface (ie, what the user sees and interacts with), including the HyperText Markup Language (HTML) (ie, the building blocks of a Web page) and the cascading style sheets (CSS) (ie, the visual formatting of the HTML). The HTML and CSS information was then interpreted by the Web browser on the user’s mobile device to create the user interface (see Figures 1-5). To illustrate an example of this programming, if a user recorded 0.5 servings of fruit, this data was stored in the relational database and the user was instantly presented with an image of half of one apple shaded green (representing 0.5 servings of fruit) on the home screen of the eVIP app (see Figure 1A). A separate app was created for each of the four behaviors, rather than creating a combined app, to permit the targeting of different behaviors in future intervention research, such as addressing only those behaviors that are particularly challenging for the individual. Further, there was a need to limit each app to < 6 screens, five of which were common to all of the apps (Figures 1-5), to simplify and increase the speed at which users could navigate their way through the apps.

### Stage 4: Testing the Prototypes

Data presented and manipulated in the prototype version of each app were crosschecked against the relational database by two authors (LH and AC) for accuracy. This involved recording intake and activity behaviors at random in each of the four apps and checking whether the data presented to the users were correct (eg, the total energy of a take-out food item) and that the data were accurately manipulated in the apps (eg, converting fruit and vegetable portions recorded into the number of equivalent servings or summing the total energy content of all sugar-sweetened drinks recorded).

Twenty-one adults aged 18 to 35 years who were participating in a weight loss trial were provided access to the apps, and were asked for their feedback on the performance of the apps as part of an online survey. This survey included two questions assessing the usability of the apps, including: “Did you have any problems downloading the smartphone apps?” and “Did you have any problems using the smartphone apps?” with three response options: “yes,” “no,” or “did not access them.” If they responded “yes,” they were then prompted in an open-ended question: “Please tell us what problems you experienced.” Two other open-ended questions asked, “How could the smartphone apps be improved?” and “Please tell us any other comments that you have*.” *Re-occurring themes in the qualitative responses to these open-ended questions were identified and summarized. Procedures for collecting this information were approved by the University of Sydney Human Research Ethics Committee (approval #13698).

## Results

The apps took 18 months to build including creation of the relational databases, exploring behavior change strategies, reviewing research with young adults on the key behaviors, creating the designs with information technology support, and testing the prototypes. Once the first app was developed, the others took less time with the physical activity app developed most rapidly. The development involved the fields of marketing, nutrition and dietetics, physical activity, and information technology. The cost of building all of the information into an app was approximately US $5000 per app—less than half the cost of mainstream commercial companies because we employed Information Technology students for the development.

The four apps were found to return the correct data to the user from the relational database and calculations performed by the apps were accurate (eg, calculating the energy, sugar, and alcohol content in 390 mL of a beverage recorded by the user). The same generic user interface (ie, what the user sees and interacts with) was used in all four apps. Figures 1-5 present two examples (A and B) for each of the five generic screens of the user interface. The first screen users see when they launch one of the apps is a log-in screen (Figure 2), that requires users to enter a unique 4-digit log-in ID code. This was to protect the privacy of users (in the case that these apps were used in future human research), as well as the intellectual copyright of the university. The second screen seen by the users after they log in shows the home screen (Figure 1), that displays a summary of the user’s behavior compared with reference guidelines. Screens presented in Figures 3 and 4 allow users to record their behaviors and review or edit data they have entered, respectively. Details about reference guidelines are then displayed in a “targets” screen (Figure 5).

Of the 21 participants offered to use these apps, only 10 evaluated them. These participants reported no difficulty with downloading the apps. Overall, participants did not like having to log in to use the apps. Some participants complained the apps operated slowly on their mobile devices (eg, “The smart phone app was a good idea but as it was a Web app, it often froze and it was a bit slow in general and scrolling lists were not functional on some operating systems” [from a 19-year-old female] and “Some parts of the apps didn’t work for me, such as the scroll, so I couldn’t enter many fruits/veg” [from a 22-year-old female]). Only one respondent (33-year-old female) provided a suggested improvement: “Applications could be designed to reward/monitor good behaviors only...”

## Discussion

In this paper, we have described the process of developing four smartphone apps aimed at improving nutrition and physical activity lifestyle behaviors during young adulthood. The apps were found to present precise data and to manipulate data accurately for the user. A small sample of young adults provided qualitative feedback. It was found that the slow running speed of the apps (because of their reliance on an active Internet connection) was an issue for the target audience (young adults aged 18 to 35), as was the requirement to log in to the apps. There was little suggestion for change in the information provided or graphics used in the apps, although most of the open-ended questions were posed to ask about issues or problems with the apps so that it generated negative rather than positive feedback. It is also acknowledged that the small sample size of participants testing the apps limits the validity of these findings.

Very few researchers in public health have reported on the development and use of smartphone apps for individual dietary or physical activity change. Mattila et al used a wellness diary for recording self-management of weight-related behaviors [34], Hughes et al developed an app for monitoring energy balance [35], Lee et al developed a weight loss diet game [36], while others have monitored diet or physical activity as part of a program for diabetes [37] or cardiac rehabilitation [38]. The uptake and usage of these apps has been moderate to high among adults in the intervention setting [34,36-38]. Smartphone apps have the potential to improve population health, largely because of their widespread and increasing use, dynamic technological advancements, ability to download updates, and use of existing features (eg, Internet access, geopositioning technology, as well as photo, video, and voice recording capabilities), and the potential for reducing intervention delivery costs. However, there are limitations to the use of smartphone apps, primarily because they may be expensive to develop and their use is often irregular and short-lived [34]. Therefore, if the target behaviors require commitment in the longer term, as is the case with nutrition and physical activity behaviors, additional support strategies may be required to prolong individuals’ motivation to use the apps [34]. Also to consider, is the competition from new apps being developed. For this reason, an audit of existing apps is recommended to inform whether adequate apps are already available [8]. A further limitation is that other barriers to nutritional and physical activity behavior change perceived by young adults cannot be addressed, such as financial costs and aspects of their social and physical environments [39,40], although one can address personal barriers, such as time constraints [39-42] or lack of self-monitoring skills [20]. The equality of using apps as a public health strategy also remains questionable, and is likely to depend on the specific target group of interest.

Although young adults are increasingly using smartphones, use in other population groups is unclear. For this reason, formative research with the target population may be required for some groups, such as older adults, before embarking on developing apps for this demographic [43]. Future research should also examine how commercially developed apps for diet or physical activity are being used by different population groups to improve our understanding of how this technology may be used to support behavior change.

The feedback from trialing the apps with the target population (young adults aged 18 to 35) will be used to refine the prototype versions of the developed smartphone apps. Attempts will be made to increase the speed of the apps and ensure functionality on all mobile phone operating systems popular with young adults. The revised apps will then be formally tested for their “usability,” which measures the ability of a software product to be understood, learned, used, and be attractive to the user, and will involve an analysis of the number of steps or time required to complete set tasks within the software [44]. Others have extended these methods to testing the usability of mobile phone apps, suggesting additional items for evaluation [45,46]. The apps will be added as part of a multi-component randomized controlled trial in young adults, together with mobile phone text messaging and phone coaching calls, to evaluate their impact using validated measures of diet, physical activity, and anthropometrics.

Smartphone apps may be an innovative medium for delivering individual health behavior change intervention en masse. Researchers or health professionals considering developing an app in their area must give careful consideration to the target population in terms of their access, ability to adopt this form of intervention, and preferences regarding the design, the current technologies available for app development, existing commercial apps, and the possibility that their use will be irregular and short-lived.

## Abbreviations

app: smartphone application
CARDIA: Coronary Artery Risk Development in Young Adults
CSS: cascading style sheets
ePASS: physical activity smartphone app
eSIYP: sugar-sweetened drinks smartphone app
eTIYP: take-out (fast food) smartphone app
eVIP: fruit and vegetable smartphone app
HTML: HyperText Markup Language
ID: identification
MET: metabolic equivalent of task
SQL: structured query language
